# Evaluation of Proanthocyanidins from Kiwi Leaves (*Actinidia chinensis*) against Caco-2 Cells Oxidative Stress through Nrf2-ARE Signaling Pathway

**DOI:** 10.3390/antiox11071367

**Published:** 2022-07-14

**Authors:** Ji-Min Lv, Mostafa Gouda, Xing-Qian Ye, Zhi-Peng Shao, Jian-Chu Chen

**Affiliations:** 1 College of Biosystems Engineering and Food Science, National-Local Joint Engineering Laboratory of Intelligent Food Technology and Equipment, Zhejiang Key Laboratory for Agro-Food Processing, Zhejiang Engineering Laboratory of Food Technology and Equipment, Zhejiang University, Hangzhou 310058, China; lvjimin@zju.edu.cn (J.-M.L.); psu@zju.edu.cn (X.-Q.Y.); 2 Department of Nutrition & Food Science, National Research Centre, Dokki, Giza 12622, Egypt; 3 School of Food Science and Biotechnology, Zhejiang Gongshang University, Hangzhou 310018, China; 15010000019@pop.zjgsu.edu.cn

**Keywords:** antioxidant response element (ARE), Nrf2 signaling pathway, bioactive byproducts, proanthocyanidins, oxidative stress mechanisms

## Abstract

Proanthocyanidins (PAs) are considered to be effective natural byproduct and bioactive antioxidants. However, few studies have focused on their mode of action pathways. In this study, reactive oxygen species (ROS), oxidative stress indices, real-time PCR, Western blotting, confocal microscopy, and molecular docking were used to investigate the protective effect of purified kiwi leaves PAs (PKLPs) on Caco-2 cells’ oxidative stress mechanisms. The results confirmed that pre-treatment with PKLPs significantly reduced H_2_O_2_-induced oxidative damage, accompanied by declining ROS levels and malondialdehyde (MDA) accumulation in the Caco-2 cells. The PKLPs upregulated the expression of antioxidative enzymes (GSH-px, CAT, T-SOD) and the relative mRNA (Nrf, HO-1, SOD-1, CAT) of the nuclear factor erythroid 2-related factor (Nrf2) signaling pathway. The protein-expressing level of the Nrf2 and its relative protein (NQO-1, HO-1, SOD-1) were significantly increased (*p* < 0.05) in the PKLPs pre-treatment group compared to the model group. In conclusion, the novelty of this study is that it explains how PKLPs’ efficacy on the Nrf2-ARE signaling pathway, in protecting vital cells from oxidative stress, could be used for cleaner production.

## 1. Introduction

With the increase in demand for natural safe alternatives to synthetic chemicals, scientists are searching for efficient techniques to evaluate the functionality of phytochemicals [1,2]. The correlation between phytochemicals and cellular antioxidant enzyme activity, based on their chemical and molecular gene expressions, can provide accurate information about their functionalities [3,4,5]. For instance, proanthocyanidins (PAs), which are abundant in kiwi (*Actinidia chinensis*) fruits and leaves, are a byproduct of kiwi fruit production, and are secondary plant metabolites that belong to the class of flavan-3-ols, with several biological activities and a wide range of health-related benefits [6,7].

For instance, as a natural safe extract, kiwi PAs have vital antioxidant, antidiabetic, and antimicrobial properties, and outstanding anticancer effects [8,9]. Our previous work found that purified kiwi leaves PAs (PKLPs) were relatively high, with a yield of 6.23% (dry weight), making kiwi leaves byproducts a commercially viable source of PAs. Moreover, the PAs isolated from kiwi leaves are mainly composed of (epi)afzelechin, (epi)catechin and (epi)gallocatechin [6]—in which regard, this structure is unique compared to other PAs’ plants sources (like baobab seeds, grape seeds, and hazelnut skin).

The bioactive potential of PAs—such as their antioxidant, antimicrobial activity—make them applicable in functional food additives for the therapeutic intervention of human disorders [2]. Thus, this novel health-related natural molecule could be used in different medicinal foods applications: for instance, the effects of acid-hydrolyzed PKLPs on the viability of Caco-2 cancer cells, which indicate that the degree of polymerization (DP) of PAs has a significant effect on cancer cells [7]. However, the key antioxidative bioactive PAs fraction mode of action still requires further study.

Therefore, it is of great significance to explore the main bioactive ingredients of PKLPs as edible and potent viable antioxidant food ingredients. Based on the mean degree of polymerization (mDP) of PAs complexes, previous study has investigated the relationship between the antioxidant activity and PAs’ structure compositions. The DP significantly affected the cellular absorption of PAs fractions. Ou and Gu [10] indicated that DP > 4 of PAs were not absorbable as a result of their large molecular dimensions. Additionally, a high DP influences the bioactivity of PAs. For instance, Li, et al. [11], reported a remarkable antiproliferative activity induction of PAs on Caco-2 cells with increasing PAs DP. Additionally, Li, Chen, Li, Liu, Liu, and Liu [11] reported that PAs’ cellular antioxidant activities on Caco-2 cells decreased with the increase of their molecular weight. Under normal conditions, there is a balance between antioxidative defense and the generation of reactive oxygen species (ROS). Oxidative stress occurs when ROS production exceeds the extent of cellular antioxidative defense [12]. The increased ROS are able to disturb barrier integrity, damage cell membranes, and enhance incidences of endotoxemia and inflammation [13]. Thus, the over-production of ROS has a deleterious effect on human health, which needs to be controlled. As PAs have a significant impact on regulating central transcription factors, they have the ability to reduce ROS’ generation of Caco-2 cells [14]. Koudoufio, et al. [15], reported that PAs have a protective impact on differentiated intestinal Caco-2/15 cells’ oxidative stress (OxS) and inflammation. They reported that PAs significantly reduced malondialdehyde, as a lipid peroxidation biomarker, and raised the relative antioxidant enzymes via increasing the ratio of the Nrf2/Keap1. The uniqueness of using Caco-2 cells in studying vital antioxidant activity has been reported by Kellett, et al. [16], who noted that, due to differences in the active membrane transport among the cell types, Caco-2-based cellular antioxidant activity measurements appeared to be a more suitable method for phytochemicals bioactivities studies compared to other cell lines, like hepatocarcinoma (HepG2) cells.

The importance of studying the nuclear factor erythroid 2-related factor 2 (Nrf2) as a cellular antioxidant marker comes from its high ability to bind with Kelch-like ECH-associated protein (Keap1, the cysteine-based mammalian intracellular sensor for electrophiles and oxidants) in the cytoplasm, under normal circumstances [17]. Upon oxidative stress, the Nrf2 migrates to the nucleus, and combines with the antioxidant response element (ARE), which can upregulate the transcription of cell defense-related genes, including drug metabolizers, detoxifying enzymes, and antioxidant proteins [18]. Several studies have reported that the Nrf2 signaling pathway plays a crucial role in the activation of cytoprotective genes in response to xenobiotics, and protecting cells against oxidative stress. However, the underlying mechanisms of the antioxidative action were unclear. In particular, the relationship between the chemical structure of PAs and their antioxidant mechanisms has not been investigated before.

Arroyave-Ospina, et al. [19], noted that intracellular antioxidant activity is an effective assay for measuring ROS and oxidative stress indices. Moreover, real-time PCR, in combination with Western blot and florescent confocal microscopy, could reflect the molecular level of the genotype and phenotype relationship, through antioxidant enzymes gene expression [20]. Additionally, molecular docking is an effective tool for investigating the best intermolecular framework between bioactive phytochemicals, cellular proteins, and biological macromolecules, in order to clarify the potential mechanisms of their interactions [21,22].

Thus, the aim of this study was to characterize kiwi leaves PAs’ viable antioxidant activity against Caco-2 cells, and to emphasize the potential pathways that cause that mode of action through their binding efficiency to the ARE from their impacts on NRF2–Keap1 complexes. This could enhance the functional application of these kinds of functional bioactive components that are produced as byproducts of leaves from kiwi fruit production. Thus, it could enhance the applicability of PAs in green and sustainable industrial applications.

## 2. Materials and Methods

### 2.1. Chemicals and Materials

RNase Free dH_2_O, ethanol (analytical grade), and acetone (analytical grade) were purchased from Macklin Biochemical Technology Co., Ltd., (Shanghai, China). Hydrogen peroxide, Nile Red, Dichlorodihydrofluorescein diacetate (DCFH-DA), and Dihydroethidium (DHE) were purchased from Sigma–Aldrich (St. Louis, MO, USA). AB-8 Macroporous resin was obtained from Solarbio Science & Technology Company (Beijing, China), and Sephadex LH-20 was purchased from GE Healthcare Bio-Sciences (Uppsala, Sweden). Dulbecco’s Modified Eagle Medium (DMEM), fetal bovine serum (FBS), penicillin/streptomycin (P/S), and phosphate-buffered solution (PBS), were supplied by Gibco company (Grand Island, NE, USA). SYBR Green PCR Master Mix and TRIzol^TM^ were obtained from Thermo Fisher Scientific, Inc., (Cleveland, OH, USA). The commercial PVDF membrane (0.45 μ, immobilon) was purchased from Solarbio Science & Technology Co., Ltd., Beijing, China.

Kiwi (*Actinidia chinensis*) leaves were collected from Zhuji farm, Shaoxing, Zhejiang Province, China, during October. Then, they were freeze-dried, and grounded into powder for extracting PAs.

### 2.2. Extraction and Purificantion of PAs from Kiwifruit Leaves

Fresh kiwifruit leaves PAs were extracted by using the optimized conditions of ultrasound-assisted extraction, following the method described by Lv, Gouda, Zhu, Ye, and Chen [6], with some modifications. Firstly, freeze-dried kiwi leaves powders (10 g) were sonicated by JY92-IIDN (Ningbo Scientz Biotechnology Co., Ningbo, China) under the following optimum conditions: 30 mL/g dry weight solvent to solid ration; 40% ultrasound-amplitude; and 70 °C sonication temperature for 15 min. Secondly, the crude PAs were extracted by 0.4 L aqueous acetone solvent (80%, *v*/*v*). Then, the acetone was removed through an evaporation process, under vacuum and 40 °C temperature, by using rotary evaporator (Dragon RE100-pro, Beijing, China). Afterwards, hexane was used to remove the non-polar components from the obtained aqueous phase. Thirdly, sugars, proteins, and pigments were removed from the extracted PAs by using an AB-8 Macroporous resin column (Solarbio Science & Technology, Beijing, China). Afterwards, to further purify the obtained PAs, according to the previous study (with some modifications) of Chai, et al. [23], the above-obtained PAs (1 g, freeze-dried) were loaded onto a Sephadex LH-20 column (GE Healthcare Bio-Sciences, Uppsala, Sweden); then, methanol (50%, *v*/*v*) was used to wash the column and remove the impurities. Subsequently, 90% methanol was used to elute the fraction A (FA) anthocyanins. After that, 50% acetone was used to collect as fraction B (FB). Both fractions (FA and FB) were freeze-dried as PKLPs.

### 2.3. Reversed-Phase HPLC-QTOF-MS/MS Analysis of PKLPs

The separation of the phenolic compounds was performed following our previous study [7]. The column used for analysis was the Luna HILIC column (Phenomenex, Torrance, CA, USA; 250 × 4.6 mm; 5 μm), with a flow rate of 0.35 mL/min at 30 °C. Injections of 10 µL of each purified extract were injected into a Waters 2489 HPLC with a UV-Vis detector (Waters Corp., Milford, MA, USA), using a mobile phase comprising a linear gradient of 99.5% acetonitrile and 0.5% acetic acid (solvent A),96.9% acetonitrile, 3% water, and 0.1% acetic acid (solvent B). The detection wavelength was set at 280 nm to monitor all phenolic compounds. For comparison, the elution conditions for solvent B were: 0–10 min, 15%; 10–20 min, 15–20%; 20–70 min, 20–60%; and 70–80 min, 60–100%. The separated compounds were fractionated and defined by mass spectra, using a Triple-TOF 5600+ ion trap mass spectrometer (AB scientific, Framingham, MA, USA). Three replicates of each sample were collected for data analysis.

### 2.4. Cell Culture and Treatment

The Caco-2 (human colonic carcinoma) cell line was provided by the Cell Resource Center, Shanghai Institutes for Biological Sciences, Chinese Academy of Sciences. The cells were cultured at 37 °C under 5% CO_2_, in a DMEM medium (Gibco) with 1% P/S (Gibco) and 20% FBS (Gibco). The cells were sub-cultured 3–4 times a week, and replaced in a fresh medium to keep the cells in a good growth state.

### 2.5. Injured Cell Model Induced by H_2_O_2_

The antioxidant activity of the H_2_O_2_-induced cell death of the Caco-2 cells was determined according to a previous study [24]. Briefly, the Caco-2 cells were seeded in cell culture 96-well plates, with a density of 1 × 10^5^ cells/mL, and cultivated for 24 h at 37 °C. Afterwards, the medium was removed, and the wells were washed 3 times with PBS, before a new, fresh medium (containing 200 μM/L H_2_O_2_) was added. The group treated by the same cell medium without H_2_O_2_ was taken as the control group. The H_2_O_2_ treatment lasted for 4 h, and the results were expressed as cell viability [7].

### 2.6. Intracellular Antioxidant Activity Assay

The effect of PKLPs’ antioxidant activity on the Caco-2 cells was determined according to previous literature, with some modification [25]. Briefly, the Caco-2 cells were seeded in cell culture 96-well plates, with a density of 1 × 10^5^ cells/mL, and cultivated for 24 h at 37 °C. The experimental groups were treated with various concentrations of samples (50 μg/mL of FA; 50 μg/mL of FB; 75 μg/mL of catechin; each for 100 μL) 24 h before H_2_O_2_ treatment. The control group was normally cultivated without H_2_O_2_ or PAs, and the model group was only treated with H_2_O_2_ to cause the oxidant damage. The result was expressed as the cell viability determined by the cell counting kit (CCK-8 assay), as described in the literature [7].

### 2.7. Determination of Reactive Oxygen Species (ROS)

The cellular ROS was determined according to previously described methods, with slight modifications [26]. Briefly, Caco-2 cells were seeded into 12-well plates, at a density of 1 × 10^5^ cells/mL, for 24 h cultivation at 37 °C. The experimental groups were treated with various concentrations of samples (50 μg/mL of FA; 50 μg/mL of FB; 75 μg/mL of catechin; each for 100 μL) for 24 h cultivation, followed by treatment with (200 μM/L) H_2_O_2_ for another 4 h. The control group was normally cultivated at the same time, and the model group was only treated with (200 μM/L) H_2_O_2_ for 4 h. After incubation with 10 μM dichlorofluorescein diacetate (DCF-DA) at ambient temperature in the dark for 30 min, the cells were instantly washed 3 times by PBS. And then, the fluorescence value was measured by the fluorescence microscope (Nikon, Tokyo, Japan) at an excitation wavelength of 485 nm, and an emission wavelength of 525 nm. The fluorescence intensity was calculated by image analysis software ImageProPlus 6.0 (Media Cybernetics, Inc., Rockville, MD, USA), and expressed as mean DCF fluorescence intensity.

### 2.8. Determination of Oxidative Stress Indices

Malondialdehyde (MDA, #S0131M), catalase (CAT, # S0051), total superoxide dismutase (T-SOD, #S0101S), and glutathione peroxidase (GSH-Px, #S0056), were measured by the commercial kits purchased from Beyotime Biotechnology Company (Shanghai, China), according to the manufacturer’s instructions.

### 2.9. RNA Extraction, Reverse Transcription, and Quantitative Real-Time PCR

Total RNA was extracted from the cells using TRIzol^TM^ (Thermo Fisher Scientific, Inc.), and diluted to 1 μg/μL. qPCR was performed according to a previously described method. Firstly, cDNA was synthesized from 1.0 μg of total RNA using the PrimeScript RT reagent Kit (TaKaRa, Japan), in a final volume of 20 μL with 10 μL master mix (4 μL RNase Free dH_2_O, 4 μL 5× PrimeScript Buffer, 1 μL RT Mix, and 1 μL PrimeScript RT Enzyme Mix). Then, 1 μL of cDNA template, 1 μL of upstream and downstream primers, 10 μL SYBR Green PCR MasterMix, and 7 μL of RNase Free dH_2_O were mixed, before being carried out in a Stepone Plus qPCR instrument (Thermo Fisher Scientific, Inc.). The PCR conditions were set as: 95 °C maintained for 5 min, followed by 40 cycles of 95 °C for 15 s and 60 °C for 40 s. The primer sequences are listed in Table 1.

Relative mRNA expression was normalized to the control group. The 2 ^−^^ΔΔCt^ formula was used to quantify, using *β-actin* as a reference gene [27]. All the results were obtained from at least three independent experiments.

### 2.10. Immunofluorescence

Samples were blocked with bovine serum (5%, *w*/*v*) for 30 min at ambient temperature, and repaired with 10.2 mM sodium citrate buffer. Then, the following primary antibodies against the Nrf2 (1:200), and the HO-1 (1:200) were used for overnight incubation at 4 °C. Afterwards, the samples were washed with PBS before incubation with anti-rabbit secondary antibodies DAPI staining at ambient temperature for 1 h. The treated Caco-2 cells were visualized using a Zeiss LSM 780 confocal microscope (Carl Zeiss SAS, Jena, Germany), and the DP2-TWAN image-acquisition system (Olympus Corp., Tokyo, Japan).

### 2.11. Molecular Docking

The initial crystal structures of Keap1 and the Nrf2 (PDBID: 2FLU) were obtained from the RCSB PDB database (http://www.rcbs.org, accessed on 15 February 2022), as reported in previous literatures [28,29]. The 3D structure of the PAs was built by Chem3D Ultra 12.0, and energetically minimized with an MM2 force field. The interaction between Keap1, the Nrf2, and the PAs was investigated by using docking analysis. Schrodinger^®^ docking suits were selected for the molecular docking studies, and a Glide^®^ receptor grid generator was used to create the grid sites with default parameters. Both the protein and the ligand structures were refined using an OPLS3e forcefield, to get correct formal charges and protonation states. A receptor grid was then generated with the prepared structure, with correct formal charges and protonation states. Finally, the ligand was docked into the corresponding protein structure. After docking, the results were ranked according to a scoring function, combining GlideScore with Prime energies, and the complex features of the protein-ligand were visualized with Pymol (Delano Scientific LLC, San Carlos, CA, USA).

### 2.12. Western Blot

Western blot was conducted, following the method of Su, et al. [30]. In brief, the cytoplasmic protein was separated by NE-PER kit (n. 78833, Thermo Fisher Scientific, Rockford, IL, USA). The separated protein contained 1% phenylmethanesulfony fluoride (PMSF, ST506, Beyotime Biotech, China) for preventing the degradation of protein, and the concentrations were measured using the bicinchoninic acid method (BCA, P0012, Beyotime Biotech, China). The separated protein was produced by electrophoresis on SDS-polyacrylamide gels, then transferred to 0.45 μm polyvinylidene fluoride (PVDF) membranes (immobilon, Solarbio Science & Technology Co., Ltd., Beijing, China). After blocking with 5% non-fat dry milk, in PBS containing 0.1% Tween-20, the membrane was incubated with the primary antibody at 4 °C for 14 h. Then, the membrane was incubated with horseradish peroxidase-conjugated secondary antibodies (ab97205 and ab97215, Abcam, Cambridge, MA, USA) for 1–2 h after washing 3 times. Next, a chemiluminescent HRP substrate was used to visualize the immunoreactive protein bands (Millipore, WBKLS0100), after washing 3 times. Primary antibodies were used as follows: the nuclear factor erythroid 2-related factor 2 (Nrf2) for humans (ab62352), heme oxygenase-1 (HO-1) for humans (ab13243), and Kelch-like ECH-associated protein 1 (Keap1) for humans (ab139729) were procured from Abcam (Cambridge, MA, USA); superoxide dismutase 1 (SOD-1) for humans (sc-17767), quinone oxidoreductase 1 (NQO1) for humans (sc-271116), and B-cell lymphoma 2 (Bcl-2) for humans (sc-7382), were procured from Santa Cruz Biotechnology Inc. (California, MA, USA).

### 2.13. Statistical Analysis

Experiments were conducted in triplicate, and the results were presented as mean ± standard deviation. Data were further analyzed via one-way analysis of variance (ANOVA), using SPSS 19.0 (Chicago, IL, USA). Duncan and Least Significant Difference (LSD) analyses at *p* < 0.05 level were used to significantly differentiate the studied treatments. IC_50_ was calculated based on the regression equation.

## 3. Results and Discussion

### 3.1. Comparation of the Chemical Composition of PKLPs (FA and FB) by Reversed-Phase HPLC-QTOF-MS/MS

**Figure 1:**
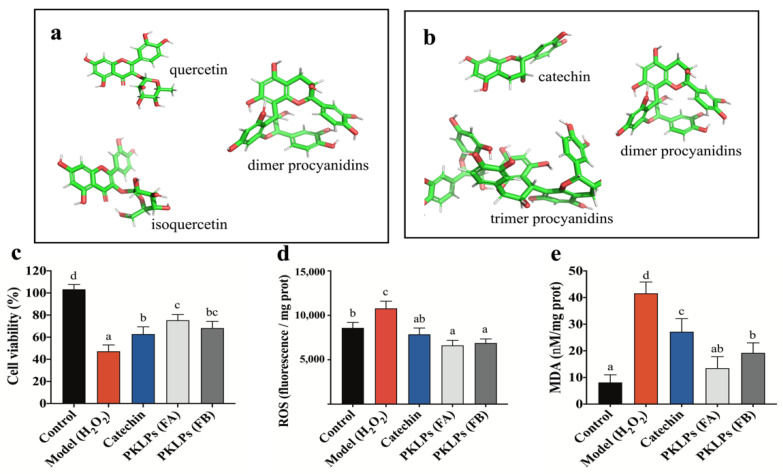
Chemical structure of eluted fractions: (**a**) FA; (**b**) FB. Enhancement of cell viability and protection of Caco-2 cells from H_2_O_2_-induced oxidant damage by pre-treatment with PKLPs: (**c**) cell viability; (**d**) intracellular generation of ROS; (**e**) malondialdehyde (MDA) level of Caco2 cells. The reported values are represented as mean ± SD (*n* = 3). Columns marked with different lowercase letters indicate the significant differences among treatments by using Duncan analysis.

According to our previous study [7], FA contains small molecule flavonoids, including quercetin, isoquercetin, and polyphenols, such as procyanidins (Figure 1a). In the present study, we focused on analyzing the composition of PAs fractions without the acid hydrolysis post-purification process. In addition, we marked the retention time (Rt) of PAs with different degrees of polymerization. We also marked the main ionized fragments of PAs in QTOF-MS^2^: molecular ions at *m*/*z* 447, with major fragment ions at *m*/*z* 301 ([M − H − 146]^−^) and *m*/*z* 109 ([M − H − 338]^−^), which were identified as quercetin based on their fragmentation patterns and retention time (Table 2). According to the results of QTOF-MS^2^, the FA included one molecular ion peak [M − H]^−^ at *m*/*z* 463, with ionic fragmentation MS^2^ at *m*/*z* 301 and 463, which was consider as isoquercetin. Fraction A contained catechin, epicatechin, and procyanidins, with its molecular ion peak ([M − H]^−^) at *m*/*z* 289 and 577, respectively, while tandem mass spectrometry yielded typical fragment ions at *m*/*z* 163, 137, 245, and 287, 289, 425, 451, respectively. The ionic fragmentations at *m*/*z* 163 and 451 ([M − H − 126]^−^) resulted from the loss of a neutral molecule, A-ring (1,3,5-trihydroxybenzene, 126 Da), through heterocyclic ring fission (HRF). The typical fragment ions at *m*/*z* 137 and 425 derived from retro-diels-alder (RDA) reaction through the loss of a neutral fragment containing the B-ring (152 Da) [31].

Fraction B contained monomer procyanidins and polymer PAs, such as catechin, epicatechin, procyanidins, prodelphindins, and propelargonidins (Figure 1b). For instance, FB showed the fragment ion ([M − H − 288]^−^) at *m*/*z* 289 and 577, that derived from cleavage of the trimer procyanidins; it also contained one molecular ion peak [M − H]^−^ at *m*/*z* 865, with MS^2^ yielding typical fragment ions at *m*/*z* 577, 289, 739, and 713. The base ion [M − H − 126] at *m*/*z* 739 came from HRF. The ion of *m*/*z* 713 was formed through an RDA fission, which characterizes hydroxvyinylbenzenediol elimination ([M − H − 152]^−^). Compared with FA, the chemical composition of FB was purer. The concentration of procyanidins as extension units in FB was much higher than that in FA, as well as the quantity of monomers units in FB. On the other hand, there was a significant difference in the DP of the two fractions. Specifically, the mean degree of polymerization (mDP) values of the eluted fractions of FA and FB were 3.2 and 5.9, respectively, which implied that FA had a smaller molecular weight than FB.

### 3.2. PKLPs (FA and FB) Suppressed H_2_O_2_-Induced Oxidative Stress in Caco-2 Cells

As hydrogen peroxide (H_2_O_2_) was a product of the cellular oxygen metabolism that was a feature of the various metabolic and signaling cascades [12,32], the control of the physiological H_2_O_2_ intracellular concentration, as an antioxidant indicator, has a significant relationship to the cells’ functionality and viability [33]. Therefore, the potential antioxidant activity of PKLPs on the cellular H_2_O_2_-induced oxidation model was established. In our previous study, we found that using 200 microM of H_2_O_2_ showed the most suitable impacts on Caco-2 cells, compared to 10, 25, 50, and 100 μM, which each caused too low an injury on the cells [6]. Therefore, 200 μM H_2_O_2_ was selected for the injury cell model.

**Figure 2:**
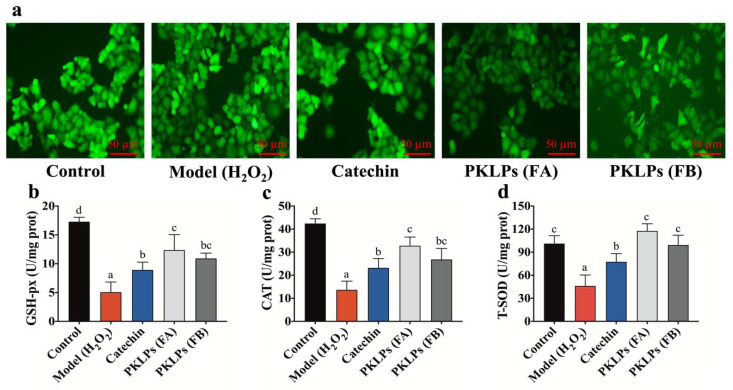
Effects of PKLPs on: (**a**) ROS level express mean fluorescence intensity; (**b**) GSH-px; (**c**) CAT; (**d**) T-SOD activities in H_2_O_2_-induced Caco-2 cells. The cells were pre-treated with catechin (75 mg/mL) and PKLPs (50 mg/mL) before being stimulated with H_2_O_2_ (200 μM/L) for 4 h. The reported values are represented as mean ± SD (*n* = 3). Columns marked with different lowercase letters indicate the significant differences among treatments by using Duncan analysis (*p* < 0.05).

In this study, a significant inhibition (*p* = 0.01; 47.25 ± 5.72%) was observed after using 200 μM of H_2_O_2_ on treating Caco-2 cells for 4 h. On the other hand, pre-treatment with PKLPs observably increased the Caco-2 cell viability after using the same concentration of ROS. Meanwhile, FA cell viability (73.16 ± 7.27%) was higher than FB, with cell viability of 69.10 ± 7.31% (Figure 1c). This phenomenon was confirmed by the fluorescence microscope, which clearly defined the increase in the number of viable cells by FA pre-treatments compared to FB (Figure 2a).

The distinction between FA and FB came from the chemical structure of PKLPs with a higher DP of FB, which decreased its functionality [15]. Rauf, et al. [34], reported that extracted PAs revealed effective antioxidant activity as a result of their functional hydroxyl groups positions, compared to other phenolic compounds. Additionally, Koudoufio, Feldman, Ahmarani, Delvin, Spahis, Desjardins, and Levy [15] mentioned that pretreatment with PAs could significantly prevent the Caco-2/15 cell from oxidative damage to its biological macromolecules due to its exposure to the strong oxygen-radicals (Fe/Asc).

For the cytoprotective effect, the two fractions of PKLPs were evaluated for their intracellular free radicals scavenging ability on the ROS and MDA cellular levels (Figure 1d,e): the ROS levels reflected the antioxidant enzyme activity, and the MDA level reflected the cellular lipid oxidation degree that was damaged by the excessive exposure to free radicals [35,36].

This study shows that ROS levels significantly (*p* = 0.03) declined from 10.8 ± 0.8 × 10^3^ florescence/mg protein in the model group to 6.6 ± 0.6 × 10^3^ in the FA group (Figure 1d). Meanwhile, the Caco-2 treated with FB showed a significant decrease (*p* = 0.04) in the release of ROS compared to the normal cells (Figure 1d). Furthermore, the MDA of FB decreased (*p* < 0.05) to 18.58 ± 3.34 nM/mg protein compared to the model with 41.59 ± 4.23 nM/mg protein. Zhou, et al. [37], reported that PAs contain an abundance of hydroxyl groups, and can release H^+^ to form cross-linkages with the radicals, which can significantly attenuate oxidative damage, by reducing the contents of MDA and ROS. Furthermore, Koudoufio, Feldman, Ahmarani, Delvin, Spahis, Desjardins, and Levy [15] reported that PAs can decrease lipid peroxidation through their ability to regulate the expression of TNFα, COX2, and NF-κB. On the other hand, Li, Liu, Li, McClements, Fu, and Liu [8] reported that reduction of the influence of PAs on Caco-2 cells viability is dependent on the specific mDP fractions that stimulate the apoptotic pathways of caspase-9, caspase-3, and caspase-8, which are generally increased by ROS generation. They also noted that the bioavailability of PAs can be increased through the hormesis effect, if it is under a lower mDP. Therefore, the study of PAs’ specific fraction vital influence should be emphasized.

### 3.3. Protective Effect of PKLPs (FA and FB) in H_2_O_2_-Induced Caco-2 Cells

Glutathione peroxidase (GSH-Px) activity, catalase (CAT) activity, and total superoxide dismutase (T-SOD) activity are known to be a crucial enzyme-driven antioxidant defense system in organisms, which can scavenge free radicals to maintain the redox balance in cells [38]. As catechin has significant antioxidant activity, as reported previously, we selected it for the positive treatment group. In this study, there was a significant increase in the antioxidant enzyme biomarkers of the PKLPs compared to the oxidative stress model, in which regard, pre-treatment of H_2_O_2_-induced cells with PKLPs (FA or FB) significantly promoted GSH-Px activity when compared to the model group (Figure 2b). Meanwhile, FA exhibited a more noteworthy effect on enhancing antioxidant enzyme activity than FB, which significantly increased to 12.36 ± 2.70 U/mg protein in the FA group, and increased to 10.91 ± 0.93 U/mg protein in the FB group, respectively, when compared to the model group (4.39 ± 1.41 U/mg protein) (*p* < 0.05). Fujimaki, et al. [39], noted that PAs contain (+)-catechin as an upper unit that mainly increases its bioactivity in its unique structural combination with (−)-epicatechin-(4β→8)-(−)-epicatechin 3-*O*-gallate subunits. Moreover, Caco-2 treated with FB increased the release of CAT significantly (*p <* 0.05), to 23.21 ± 4.05 U/mg protein compared to the model group (Figure 2c).

As shown in Figure 2c,d, CAT and T-SOD activities in the PKLPs treatment group exhibited similar antioxidant activity to GSH-Px activity. From the results, we found that PKLPs pre-treatment could effectively improve the antioxidant status of cells exposed to free radicals damage by increasing the GSH-Px, CAT, and T-SOD activities, which indicated that PKLPs could activate an antioxidant signaling pathway [34]. Our findings are consistent with the previous study of Su, Li, Hu, Xie, Ke, Zheng, and Chen [30]: PAs can attenuate oxidative stress by scavenging excessive ROS, and protect the organism by increasing the activity of antioxidant enzymes.

### 3.4. Effect ofPKLPs (FA and FB) on the Nrf2 and Its Downstream Target Genes Transcription

The employment of micro(m)RNA expression data as molecular markers could explain the antioxidant and anticancer activities potential pathway through the transcription factors [40]. For instance, the nuclear factor erythroid 2-related factor (Nrf2) is a transcription factor that plays a significant role in response to xenobiotics and oxidative stress, by binding to the antioxidant response element (ARE) [41].

**Figure 3:**
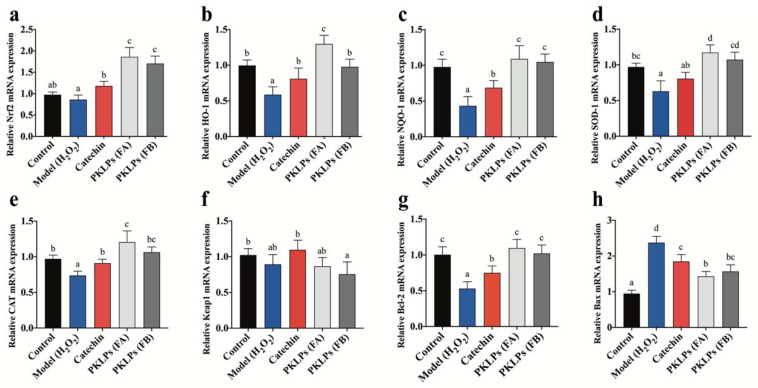
Effects of PKLPs on Nrf2-mediated antioxidants signaling transcripts were analyzed by qRT-PCR. The gene expression of the Nrf2 and its relative or downstream molecules: (**a**) Nrf2 mRNA; (**b**) HO-1 mRNA; (**c**) NQO1 mRNA; (**d**) SOD-1 mRNA; (**e**) CAT mRNA; (**f**) Keap1 mRNA; (**g**) Bcl-2 mRNA; (**h**) Bax mRNA. Data are shown as mean ± standard deviation (*n* = 3). Columns marked with different lowercase letters indicate the significant differences among treatments by using Duncan analysis (*p* < 0.05).

In this study, the mRNA levels of the Nrf2 and Keap1, and the downstream target genes, were further investigated in Caco-2 cells for their potential response to various treatments. Quantitative real-time PCR (qRT-PCR) analysis of relative mRNA expression is shown in Figure 3. There were significant increases in the antioxidant mRNA biomarkers of the PKLPs pre-treated groups compared to the model group, in which regard, the Nrf2 levels were significantly increased to 1.86 ± 0.22 mRNA expression in the FA group compared to the catechins with 1.18 ± 0.11 mRNA expression (Figure 3a). Meanwhile, the Caco-2 treated with FA increased the release of HO-1 significantly (*p =* 0.03), to 1.29 ± 0.12 mRNA expression, compared to FB, with 0.97 ± 0.11 mRNA expression (Figure 3b), in which regard, the changes in FA and FB that impacted on the mRNA antioxidant expression could be ascribed to the higher molecular weight of FB, which may have blocked its accessibility into the Caco-2 cell, and therefore decreased its impacts [10] (Figure 3c). Ge, et al. [42], reported that PAs’ impact on Caco-2 cell monolayers and cholesterol could explain their different dimers’ impacts on the signaling pathways through the absorption affinity. The same trend was observed for the mRNA level of NQO1, SOD-1, CAT, and Bcl-2 (Figure 3c–e,g), while the mRNA expression of Keap1 and Bax showed different responses (Figure 3f,h). For instance, the Caco-2 pre-treated with FA significantly decreased the mRNA expression of Bax (*p* = 0.02), with 1.43 ± 0.13, compared to the model with 2.37 ± 0.18 (Figure 3h). Contrastingly, the PKLPs pre-treatment groups remarkably reversed the expression level of these genes compared to the model group, which indicates that PKLPs pre-treatment could significantly inhibit oxidative stress, by alleviating the alteration in Bcl-2 and Bax gene transcription (Figure 3g,h). Siddiqui, et al. [43], mentioned that Bax, as a pro-apoptotic protein, could promote the release of apoptotic molecules into the cytoplasm by competing with CAT, in which its expression was affected by the antioxidant molecules against ROS.

**Figure 4:**
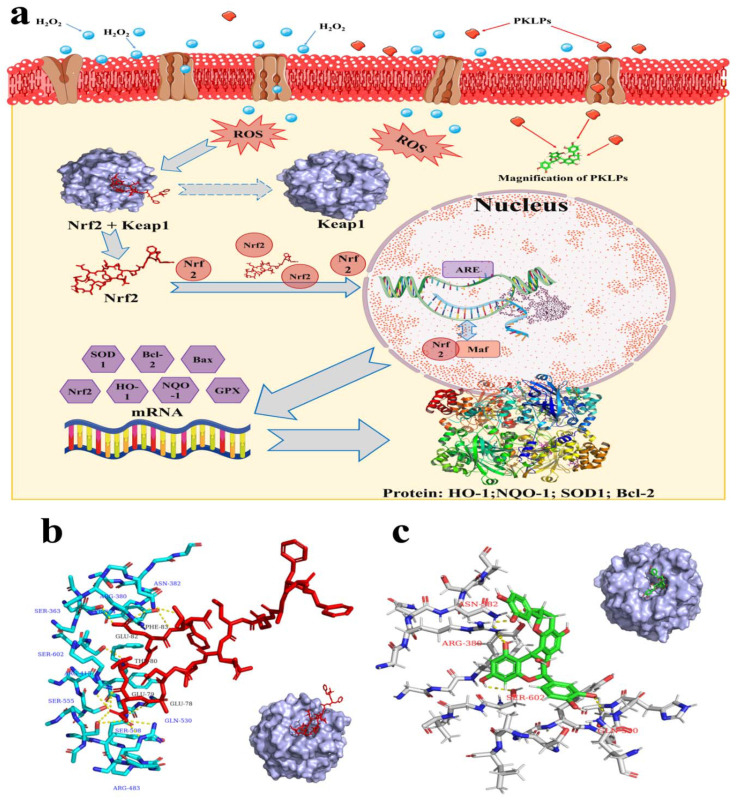
Comparison between the computational geometries of the binding of the Nrf2 and PKLPs with a pocket of Keap1 protein: (**a**) Proposed schematic diagram of the Nrf2 signaling pathway mechanism of the PKLPs in the H_2_O_2_-induced Caco-2 cells; (**b**) Magnification of the interaction site of the Nrf2–Keap1 complex motif, and its binding geometry; (**c**) Magnification of the interaction between the Keap1 pocket and the PKLPs. Hydrogen bonds are represented by yellow dashed lines, and the related amino acid residues and peptides are highlighted in the picture.

Our results are consistent with previous results, which confirmed that polyphenols extracted from plants or herb could increase antioxidative enzymes based on the upregulation of their antioxidative mRNA genes expression, such as NQO-1, HO-1, Nrf2, and SOD [44]. Hilary, et al. [45], reported that PAs’ mode of action came from their hydroxyl groups, which could direct supporting antioxidant reactions or even mediate the occurrence of oxidant events. This phenomenon could be explained as, under normal cellular condition, the Nrf2 binds to Keap1 to form suitable complexes in cytoplasm; on the other hand, ROS cause the separation of the Nrf2 from Keap1 and, as a result, the Nrf2 transfers into the nucleus; meanwhile, Keap1 degrades in the cytoplasm [46] (Figure 4a). The reason for the dissociation of the Nrf2–Keap1 complex in the cytoplasm is the sulfhydryl modification and Nrf2 phosphorylation, which results in an uncoupled Nrf2 and Keap1 [47].

### 3.5. Effect of PKLPs (FA and FB) on the Nrf2 and Its Downstream Protein Expression

**Figure 5:**
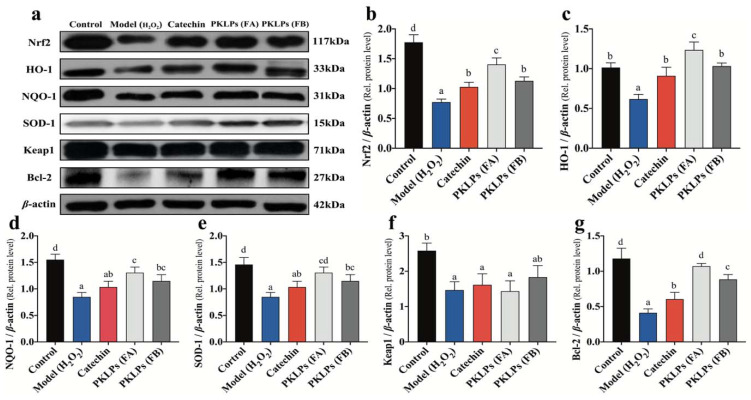
Effects of PKLPs on the protein expression of the Nrf2 signaling pathway: (**a**) Western blotting estimation of the Nrf2 signaling pathway protein expression in Caco-2 cells after pre-treatment with catechin (75 μg/mL) and PKLPs (50 μg/mL) for 24 h before stimulation with H_2_O_2_ (200 μM/L) for 2 h; (**b**–**g**) Quantification of the above-mentioned Western blots, using β-actin as a loading control. The results are presented as mean ± SD (*n* = 3). Columns marked with different lowercase letters indicate the significant differences among treatments by using Duncan analysis (*p* < 0.05).

**Figure 6:**
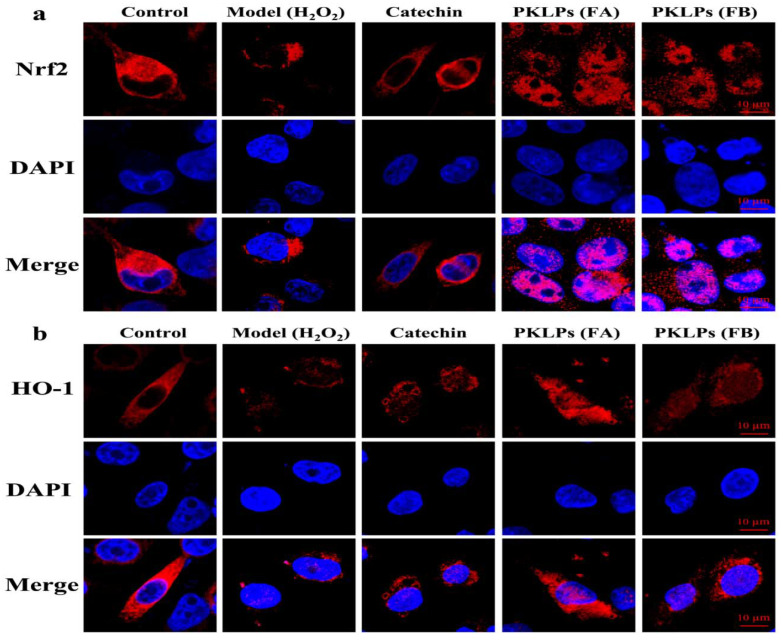
Confocal microscopy of single Caco-2 cells protein expression of (**a**) the Nrf2 and (**b**) HO-1 signaling pathways after pre-treatment with catechin (75 μg/mL) and PKLPs (50 μg/mL).

To further confirm the results of the Nrf2 and HO-1 expressions, the signaling pathway was further investigated through the molecular mechanism to underline the protective effect of PKLPs on H_2_O_2_-induced oxidative stress. The related antioxidant protein levels of the Nrf2, Keap1, SOD-1, HO-1, and NQO-1 were studied by Western blotting assay, and their changes were evaluated by confocal microscopy technology (Figure 5 and Figure 6).

As shown in Figure 5, compared to the normal control group, the antioxidant proteins were increased (*p* < 0.05) by pre-treatment of the PKLPs, when compared to the model that used the oxidative damage hydrogen peroxide as a negative control, in which regard, the Nrf2 levels were significantly increased to a 1.40 ± 0.10 Nrf2/*β*-actin relative level in the FA group, compared to the model with a 0.773 ± 0.05 Nrf2/*β*-actin relative level (Figure 5b). Additionally, the FA increased the release of HO-1 significantly (*p* < 0.05) to a 1.24 ± 0.10 HO-1/*β*-actin relative level compared to the catechins with a 0.91 ± 0.11 HO-1/*β*-actin relative level (Figure 5c). As shown in Figure 6, the confocal images showed that the distribution of the Nrf2 was increased inside the nucleus for the FA group compared to the model and catechin treated groups. It demonstrated that Nrf2 expression plays a critical role in oxidative stress response. This action is through the dissociative Nrf2 transfer from cytoplasm to nucleus, and binds to the antioxidant response element in the nucleus to exert function by activation of the gene transcription of the antioxidant enzyme [48].

Meanwhile, the obtained results revealed that the protein levels of the Nrf2 and its downstream protein were significantly increased in the FA pre-treated group, compared to those in other pre-treatment groups, such as the FB pre-treated group and the catechin pre-treated group. Based on our study, FA contained procyanidins, quercetin, and isoquercetin, while FB only contained polymer PAs. The relative antioxidative mRNA transcription and protein expression results implied that oligomer PAs and quercetin could work synergistically to regulate multiple and interactive molecular targets related to H_2_O_2_-induced oxidative damage and the involvement of Nrf2-associated pathways, which was consistent with previous reporting [30]. As for FB, the fraction had more chemical adducts that were causing bigger mDP, which might inhibit the bioavailability of PAs, and further inhibit their vital impact on enzyme activity and other bioactivities. Therefore, in this study, FA had overall activities higher than FB.

The mechanism of defense oxidative damage is clarified in Figure 4a. The insertion of FA inside the cells inhibited the ROS negative impacts, which significantly increased the excretion of Nrf2 and HO-1 antioxidant enzymes expressions. Similarly, the corresponding expression of the antioxidant enzymes (SOD-1, HO-1, NQO-1, and Bcl-2) were upregulated (*p* < 0.05) in Caco-2 cells against the H_2_O_2_ exposed group. The results of the molecular mechanisms, by qRT-PCR and the Western blot, indicated that both FA and FB of the PKLPs dramatically rescued previous effects caused by oxidative damage. H_2_O_2_ exposure inhibited the expression of the Nrf2 and its related or downstream genes such as SOD-1, HO-1, NQO-1, and Bcl-2 in organisms. Zhou, Chang, Gao, and Wang [3] reported that extracted plants’ PAs protect the organisms against apoptosis caused by H_2_O_2_, through their significant impacts on the Nrf2-ARE pathway. Furthermore, the current results agree with the previous investigations, that H_2_O_2_ treatment suppressed the translocation of the Nrf2 signaling pathway [49]. 

Molecular modelling of the interaction between Keap1, Nrf2, and PAs is shown in Figure 4b,c by using a molecular docking analysis with the binding mode of the Nrf2 to Keap1. The phenylalanine 83 (PHE-83) of the peptide was stabilized by a hydrogen bond with the asparaginate 382 (ASN-382) of the Keap1. Arginine 380 (ARG-380) and tyrosine 334 (TYR-334) from the Keap1 protein were simultaneously held together with glutamic acid 82 (GLU-82) on the Nrf2 protein by hydrogen bond. Moreover, serine 602 (SER-602) from the surface of the Keap1, was bonded to threonine 80 (THR-80) from the Nrf2 protein.

From the molecular docking simulation, it can be seen that PAs were simultaneously held together with the active cites amino acids, like aspartic acid 382 (ASN-382), arginine 380 (ARG-380), serine 602 (SER-602), and glutamine 530 (GLN-530), on Keap1 by hydrogen bonding [50]. It can be seen that PAs compete with the Nrf2 to promote the separation of the Nrf2 from the Nrf2–Keap1 complexes. The Nrf2 is then transported into the nucleus, where it binds to the antioxidant response element, and produces different antioxidant enzymes by activating relative antioxidant genes. Ma, et al. [51], noted that PAs bind with large molecular-weight proteins like β-casein through the interaction of their hydrophobic residues with the amino acid hydrophobic residues, including Ala-192, Pro-196, and Pro-201. Therefore, the increase in the affinity of carbonylic groups of the peptide backbone forming hydrogen bonds with other amino acids from Keap1 could be the best explanation of how the PAs stimulate the separation of the Nrf2 from Keap1 [47]. Under normal physiological conditions, Keap1 acts as a binder in the cytoplasm, which binds to the Nrf2 through Cul3 ubiquitin ligase, to regulate the ubiquitination of the Nrf2. As a result, the free Nrf2 is maintained at a low level in the cytoplasm.

## 4. Conclusions

Two PAs fractions (FA and FB) were considered as byproducts that were extracted from the leaves waste of kiwi fruit production. These compounds showed significant functional and vital antioxidant activity for green and sustainable production. The antioxidative mechanism of both fractions (FA and FB) was studied by using ROS measurement, oxidative stress indices, quantitative real-time PCR, Western blot analysis, confocal microscopy, and molecular docking in Caco-2 cell lines. The present study indicates that PLKPs have a potential key impact on promoting cells signaling pathways to construct the linkage between the Nrf2 and ARE in Caco-2 cells. In comparison with the models, the FA group had a more positive and significant effect compared to FB and catechin. Therefore, this study proposes the use of PAs for promoting the antioxidant capacity of life cells through enhancing antioxidant-related enzyme genes expressions; in which regard, their potential application as a medicinal bioactive ingredient in food cleaner production may enhance their lifestyle-related protective impact against oxidant-related diseases like cancers.

## Figures and Tables

**Table 1:** Primer sets for quantitative real-time PCR.

Gene	Forward Sequence	Reverse Sequence	NCBI No.
*Nrf2*	TCACACGAGATGAGCTTAGGGCAA	TACAGTTCTGGGCGGCGACTTTAT	NM_010902.4
*Keap1*	CAGCAACTCTGTGACGTGACC	TCAATAAGCCTTTCCATGACCT	NM_016679.4
*SOD-1*	TGGTTTGCGTCGTAGTCTCC	CTTCGTCGCCATAACTCGCT	NM_000454.4
*NQO-1*	GGTGAGCTGAAGGACTCGAA	ACCACTGCAATGGGAACTGAA	NM_008706.5
*HO-1*	ATGGCCTCCCTGTACCACATC	TGTTGCGCTCAATCTCCTCCT	NM_002133.2
*CAT*	CCATTATAAGACTGACCAGGGC	AGTCCAGGAGGGGTACTTTCC	NM_001752.3
*Bcl-2*	ATGTGTGTGGAGCGTCAACC	CAGAGACAGCCAGGAGAAATC	NM_000633.3
*Bax*	GAGCTGCAGAGGATGATTGCT	TGATCAGCTCGGGCACTTTA	NM_007527.3
*β-actin*	CAAGAGAGGTATCCTGACCT	TGATCTGGGTCATCTTTTCAC	NM_007393.5

**Table 2:** HPLC-QTOF-MS/MS of the different eluted fractions.

Fractions	[M − H]^−^ (*m*/*z*)	Typical MS^2^ Ions (*m*/*z*)	Molecular Formula	Compound Name	Rt (min)
*Fraction A (FA)*					
FA	289	137; 163; 245	C_15_H_14_O_6_	Catechin	8.7
FA	289	137; 163; 179; 289	C_15_H_14_O_6_	Epicatechin	11.2
FA	447	109; 301; 447	C_21_H_20_O_11_	Quercetin	13.3
FA	463	109; 301; 463	C_21_H_20_O_12_	Isoquercetin	14.7
FA	577	287; 289; 425; 451; 577	C_30_H_26_O_12_	Dimer procyanidins	18.2
FA	865	289; 577; 739; 711; 865	C_45_H_38_O_18_	Trimer procyanidins	27.1
*Fraction B (FB)*					
FB	289	137; 163; 245	C_15_H_14_O_6_	Catechin	7.7
FB	289	137; 163; 179; 289	C_15_H_14_O_6_	Epicatechin	9.7
FB	561	271; 289; 409; 561	C_30_H_26_O_11_	Dimer propelargonidins	14.1
FB	577	287; 289; 451; 425; 577	C_30_H_26_O_12_	Dimer procyanidins	16.7
FB	593	287; 305; 467; 593	C_30_H_26_O_13_	Dimer prodelphindins	21.9
FB	865	289; 577; 739; 711; 865	C_45_H_38_O_18_	Trimer procyanidins	27.5
FB	1153	289; 577; 865; 1153	C_60_H_50_O_24_	Tetramer procyanidins	37.3

## Data Availability

The data presented in this study are available in the article.

## Abbreviations

Proanthocyanidins: PAs, purified kiwifruit leaves PAs: PKLPs, fetal bovine serum: FBS, Dulbecco’s Modified Eagle Medium: DMEM, penicillin/streptomycin: P/S, phosphate-buffered solution: PBS, cell counting kit-8: CCK-8, fraction A: FA, fraction B: FB, weight basis: WD, hydrogen peroxide: H_2_O_2_, reactive oxygen species: ROS, malondialdehyde: MDA, glutathione peroxidase: GSH-px, nuclear factor erythroid 2-related factor: Nrf2, Quantitative real-time PCR: qRT-PCR, dichlorofluorescein diacetate: DCF-DA, Kelch-like ECH-associated protein 1: Keap1, heme oxygenase-1: HO-1, antioxidant response element: ARE, quinone oxidoreductase 1: NQO-1, superoxide dismutase 1: SOD-1, catalase: CAT, B-cell lymphoma 2: Bcl-2, and Bcl-2-associated X protein: Bax, HRF: heterocyclic ring fission, RDA: Retro-diels-alder, QM: quinone methide.
